# Characterization, Antioxidant Activities, and Pancreatic Lipase Inhibitory Effect of Extract From the Edible Insect *Polyrhachis vicina*

**DOI:** 10.3389/fnut.2022.860174

**Published:** 2022-04-07

**Authors:** Zongqi Zhang, Sicong Chen, Xunfan Wei, Jinhua Xiao, Dawei Huang

**Affiliations:** College of Life Sciences, Nankai University, Tianjin, China

**Keywords:** edible insect, *Polyrhachis vicina*, hydro-ethanolic extract, antioxidant activity, pancreatic lipase

## Abstract

Oxidative stress and obesity are critical risk factors for metabolic syndrome. The consumption of functional food ingredients can a viable strategy to alleviate oxidative stress and obesity. In this study, the hydro-ethanolic extract of the edible insect *Polyrhachis vicina* was prepared and its bioactive components were characterized. The total polyphenol contents, total flavonoid contents, antioxidant and pancreatic lipase (PL) inhibitory activities of the extract were determined *in vitro*. In total, 60 bioactive components were tentatively identified in the *P. vicina* extract. Polyphenols and fatty acids were further quantified using LC-MS and GC-MS, respectively. *P. vicina* extract possessed excellent antioxidant and PL inhibition activities. Salicylic acid, gallic acid, liquiritigenin, and naringenin, which were the major polyphenols in the *P. vicina* extract, interacted with PL through hydrogen bonding, hydrophilic or hydrophobic and pi-cation interactions. Thus, *P. vicina* extract can be used as a nutraceutical to alleviate oxidative stress-induced disease and manage obesity.

## Introduction

Western diet and sedentary lifestyle have contributed to the gradual increase in the incidence of metabolic syndromes, such as obesity (1). According to the World Health Organization, the prevalence of obesity has tripled from 1975 to 2016. Approximately 41 million children under the age of 6 years and 1.9 billion adults are classified as overweight or obese (2). Obesity, which is characterized by excessive accumulation of body fat and aberrant lipid metabolism, is associated with an increased risk of disability. Thus, the enzymes, including pancreatic lipase (PL), involved in lipid metabolism are potential therapeutic targets for obesity (3). Oxidative stress, a major source of free radicals, is the etiological factor for various chronic diseases, such as type 2 diabetes mellitus, cardiovascular disease, chronic inflammation, cancer and metabolic dysfunction (4). Hence, there is a need to identify effective and safe natural bioactive components to manage obesity and scavenge free radicals.

The rapid population growth and enhanced consumption of resources, as well as increased awareness of food safety and environmental pollution among consumers, have contributed to the development of traditional animal husbandry as a strategy to protect the environment. Additionally, consumers are seeking new food sources. In particular, there is increased awareness of edible insects and their applications in the preparation of food, medicines, and healthcare products. Edible insects, which have high nutritional and functional values with anticancer, anti-inflammatory, antidiabetic and antiobesity, are considered to be more environmentally friendly than livestock for the preparation of food, medicines, and healthcare products (5, 6). Additionally, various international agencies, such as the United Nations Food and Agriculture Organization consider insects as a cheap and major source of dietary protein for humans in developing countries and as a healthy food in developed countries (7).

Limited studies have identified specific bioactive compounds in edible insects. However, some edible insect-derived bioactive compounds are reported to exhibit various biological activities, including antioxidant, antitumor, antimicrobial, and anti-obesity activities (6). Liu et al. (8) demonstrated the antioxidant effect of polyphenolic compounds derived from the extracts of *Holotrichia parallela* Motschulsky. Chen et al. (9) demonstrated the potent antioxidant and antibacterial activities of chitosan obtained from *Periplaneta americana*. Ahn et al. (10) reported that a glycosaminoglycan extracted from *Bombus ignitus* queen exerted therapeutic effects on fatty liver or hyperlipidemia. Several studies have reported the therapeutic effects of the extracts of different insect species on obesity or related diseases (6). However, the specific bioactive components of the extracts have not been identified.

*Polyrhachis vicina* (Hymenoptera: Formicidae, also called black ant) has been used in traditional Chinese medicine as a nutrient and medicine since ancient times (11). The bioactive components of *P. vicina* are reported to comprise linoleic acid, heptadecenoic acid, and octadecenoic acid (12). However, limited studies have examined the phenolic components of *P. vicina*. Previous studies have reported that *P. vicina* extracts exert hyperuricemia and exhibit anti-inflammatory and antitumor activities (12, 13). Thus, the practice of *P. vicina* supplementation in wine has continued for many years in China or certain Western countries. Distilled liquor flavored with ants was believed to be highly effective against rheumatism and gout. Ant spirits, a healthy liquor, was mentioned as a pharmaceutical product in 1698 (14).

This study aimed to prepare the hydro-ethanol extracts of the edible insect *P. vicina* and determine their physicochemical parameters and bioactive functions. The bioactive compounds of the *P. vicina* extract were analyzed using an ultra-performance liquid chromatograph-Q-extractive orbitrap mass spectrometer system (UPLC-Q-Extractive Orbitrap MS). Polyphenols and free fatty acids were quantified using the liquid chromatography-tandem mass spectrometry (LC-MS) and gas chromatography-tandem MS (GC-MS). Additionally, the antioxidant and PL inhibitory activities of the *P. vicina* extract were examined. Finally, molecular docking of PL and major polyphenols of the extract was performed.

## Materials and Methods

### Chemicals and Materials

*Polyrhachis vicina* was purchased from Shanxi Shengliyuan Biotechnology Co., Ltd. (Shanxi, China). All insects were allowed to fast for approximately 48 h under suitable conditions to promote the clearance of residual food from the gastrointestinal tract. The insects were freeze-dried and ground to a powder, which was stored at −20°C until analysis. Methanol, acetonitrile, porcine PL, Dulbecco’s Phosphate-Buffered Saline were obtained from Sigma-Aldrich Chemie GmbH (Steinheim, Germany). All other chemicals used in this study were of analytical grade and purchased from Shanghai Yuanye Biotechnology Co., Ltd. (Shanghai, China) and Sinopharm Chemical Reagent Co., Ltd. (Shanghai, China).

### Preparation of Hydro-Ethanol Extract

*Polyrhachis vicina* powder (10 g) was extracted with 200 mL of 80% ethanol in an ultrasonic microwave (XH-300A, Beijing, China). The extraction conditions were as follows: ultrasonic power, 350 W; microwave temperature, 70°C; time, 30 min; number of repetitions, 3. The samples were centrifuged at 6,000 rpm for 20 min using a centrifuge (Centrifuge 5804, Eppendorf, Hamburg, Germany). Ethanol in the supernatant was vacuum-dried using a rotary evaporator, while the aqueous fraction was lyophilized for use in the ethanol extract.

### Evaluation of Physicochemical Parameters

The physicochemical parameters, including moisture, protein, lipid, total sugar, and ash contents, of the *P. vicina* extract were determined following standard chemical composition analysis methods of the Association of Official Analytical Chemists. The total phenolic content (TPC) and total flavonoid content (TFC) were determined by Folin-Ciocalteu method and Sodium nitrite-aluminum nitrate colorimetric method, respectively.

### Analysis of Bioactive Compounds in the *P. vicina* Extract

The extracts were subjected to chromatographic analysis using a Thermo Ultimate 3000 LC system equipped with a Zorbax Eclipse C18 column (100 mm × 2.1 mm, 1.8 μm, Agilent Technologies) maintained at 30°C. The chromatographic conditions were as follows: auto-sampler temperature, 4°C; mobile phase, 0.1% formic acid in water (solvent A) and acetonitrile (solvent B); flow rate, 0.3 mL/min; injection volume, 2 μL. The analyte was eluted with a linear gradient of solvent A (v/v) under the following conditions: 0–2 min, 5% solvent B; 2–6 min, 5–30% solvent B; 6–7 min, 30% solvent B; 7–12 min, 30–78% solvent B; 12–14 min, 78% solvent B; 14–17 min, 78–95% solvent B; 17–20 min, 95% solvent B; 20–21 min, 95–5% solvent B; 21–25 min, 5% solvent B. The electrospray ionization MS (ESI-MSn) experiments were performed using the Thermo Scientific™ Q Exactive™ HF mass spectrometer (Orbitrap MS, Thermo Fisher Scientific) under the following conditions: spray voltage, 3.5 and −3.5 kV in positive and negative modes, respectively; sheath gas flow rate, 45 arbitrary units; auxiliary gas flow rate, 15 arbitrary units; capillary temperature, 330°C; scanned mass range, m/z 100–1500; mass resolution, 120000. Data-dependent acquisition (DDA) MS/MS experiments were performed with a high energy collision dissociation scan at an MS/MS resolution of 60000. The S-lens radiofrequency levels of positive and/or negative modes were 55%.

The quantification of 14 polyphenols in *P. vicina* extract was performed using LC-MS analysis with the Waters ACQUITY ultra-performance liquid chromatography (UPLC) system equipped with an ACQUITY UPLC BEH C18 column (100 mm × 2.1 mm, 1.7 μm, Waters) and an AB 4000 triple quadrupole mass spectrometer (Waters, Massachusetts, United States). The UPLC conditions were as follows: mobile phase, 0.1% formic acid in water (solvent A) and methanol (solvent B); injection volume, 5 μL; flow rate, 0.25 mL/min. The gradient conditions were as follows: 0–1 min, 10% solvent B; 1–3 min, 10–33% solvent B; 3–10 min, 33% solvent B; 10–15 min, 33–50% solvent B; 15–20 min, 50–90% solvent B; 20–21 min, 90% solvent B; 21–22 min, 90–10% solvent B; 22–25 min, 10% solvent B.

### Quantification of Fatty Acid in *P. vicina* Extract

The fatty acid composition was analyzed using GC-MS by quantifying the levels of fatty acid methyl esters (FAMEs). Briefly, 0.5 g of sample was dissolved in 1 mL chloroform-methanol (2:1) and 2 mL of methanol sulfate (1%) and incubated at 80°C for 30 min. The mixture was allowed to reach room temperature (25°C) and centrifuged at 12,000 rpm and 4°C for 10 min. Next, the precipitate was incubated with n-hexane (1 mL) and distilled water (5 mL) to obtain the products. Methyl salicylate was used as an internal standard. Finally, the excess water was removed by adding anhydrous sodium sulfate (100 mg). The methyl esters were further dissolved in methylene chloride for GC-MS analysis. The analysis of FAMEs was performed using a Trace 1310-ISQ 7000 gas chromatograph (Thermo Fisher Scientific, Massachusetts, United States) equipped with a TG-FAME capillary column (50 m × 0.25 mm, 0.20 μm, Thermo). The GC conditions were as follows: carrier gas, helium; flow rate, 0.63 mL/min; injection volume, 1 μL. The sample was injected at a ratio of 8:1 into the capillary column. The GC oven temperature conditions were as follows: initially set to 80°C for 1 min and then increased at the rate of 20°C/min to 160°C; hold for 1.5 min; 3°C/min to 196°C; hold for 8.5 min; 20°C/min to 250°C, hold for 3.0 min. Meanwhile, the temperatures of the inlet system and ion source were 250 and 230°C, respectively. The scan mode and electron energy were SIM and 70 eV, respectively. FAMEs were detected using the mass spectrum database (NIST MS Library) according to the retention times of the standards.

### Antioxidant Activities of *P. vicina* Extract

#### Antioxidant Activity Analysis of the Extract Using the 2,2-Diphenyl-1-Picrylhydrazyl Assay

Different concentrations (0.01–4.00 mg/mL) of the extracts (1.0 mL) were prepared in deionized water and incubated with a DPPH⋅ methanol solution (2 mL, 0.2 mM) at 25°C for 20 min. The absorbance of the mixture at 517 nm was determined. Vitamin C (Vc) and *P. vicina* powder, which were prepared in deionized water following the same procedure, served as controls. The half-maximal inhibitory concentration (IC_50_) (mg/mL) was the effective concentration at which the extract scavenged 50% of DPPH⋅ as interpolated from linear regression analysis. The DPPH⋅ scavenging activity was calculated as follows:


$${\text{DPPH}}^{•}\mathrm{scavenging}\mathrm{activity}\left(\%\right)=\left[1-\frac{{\text{A}}_{1_{}}-{\text{A}}_{2}}{{\text{A}}_{0}}\right]\times 100$$

where A_1_ is the absorbance of the test sample; A_2_ is the absorbance of the sample without the DPPH⋅ solution, and A_0_ is the absorbance of the blank (without samples).

#### Analysis of 2,2’-Azino-Bis(3-Ethylbenzothiazoline-6-Sulfonic Acid) Radical Scavenging Activity

Briefly, 5.0 mL of ABTS (7.0 mM) was incubated with 88 μL of potassium persulfate (2.5 mM) for 12–16 h at room temperature in the dark. The absorbance of the reaction mixture at 734 nm was adjusted to 0.7 ± 0.02 using 0.1 M phosphate buffer (pH 7.4). Next, the samples (0.5 mL) were incubated with 2.0 mL of the ABTS solution for 30 min at room temperature. IC_50_ (mg/mL) was defined as the effective concentration at which the extract scavenged 50% ABTS⋅. The absorbance of the reaction mixture at 734 nm was compared with that of the blank. Vc and *P. vicina* powder reaction mixtures served as controls. The ABTS⋅ scavenging activity was calculated as follows:


$${\text{ABTS}}^{•}\mathrm{scavenging}\mathrm{activity}\left(\%\right)=\left[1-\frac{{\text{A}}_{1_{}}-{\text{A}}_{2}}{{\text{A}}_{0}}\right]\times 100$$

where A_1_ is the absorbance of the test sample; A_2_ is the absorbance of the sample without the ABTS⋅ solution, and A_0_ is the absorbance of the blank (without samples).

#### OH⋅ Scavenging Activity

Different concentrations (0.1–4 mg/mL) of samples (1.0 mL) were prepared in 0.75 mM phenanthroline (PHEN) and 0.2 M PBS solution (pH 7.4). The reaction mixture was incubated with 0.75 mM FeSO_4_ solution and 0.025% H_2_O_2_ at 37°C for 1 h. Vc and *P. vicina* powder reaction mixtures were used as controls. The absorbance of the reaction mixture at 536 nm was immediately recorded. The OH⋅ scavenging activity was calculated as follows:


$${\text{OH}}^{•}\mathrm{scavenging}\mathrm{activity}\left(\%\right)=\left[\frac{{\text{A}}_{1_{}}-{\text{A}}_{0}}{{\text{A}}_{2_{}}-{\text{A}}_{0}}\right]\times 100$$

where A_1_ is the absorbance of the test sample; A_2_ is the absorbance of the sample without the H_2_O_2_ solution, and A_0_ is the absorbance of the blank (without samples).

#### Fe^3+^ Reducing Antioxidant Power

Different concentrations of the samples (1.0 mL) were incubated with 0.2 M phosphate buffer solution (pH 6.6) and 1.0% potassium ferricyanide solution at 50°C for 20 min, followed by incubation with 10% trichloroacetic acid (TCA). The mixture was centrifuged at 3,000 rpm for 10 min. Next, 2.5 mL of the supernatant was transferred to a tube comprising 2.5 mL distilled water and 0.1% ferric chloride. The absorbance of the reaction mixture at 700 nm was measured. The experiments were repeated thrice.

### Pancreatic Lipase Inhibition Assay

#### Determination of Pancreatic Lipase Inhibition

The PL inhibitory activity of the extract was determined using a previously reported method with minor modifications (15). Orlistat and *P. vicina* powder were used as controls, while 4-methylumbelliferyl oleate (4-MUO) was used as a substrate. PL (0.1 g) was dissolved in 100 mL PBS, stirred for 20 min, and centrifuged at 8,000 rpm for 10 min. Different concentrations of extract solution (25 μL) were incubated with 25 μL of PL solution and 50 μL of 4-MUO solution for 20 min at 37°C with or without shaking. The reaction was stopped by adding 100 μL of 0.1 mol/mL sodium citrate solution. Next, 150 μL of the reaction mixture was transferred to the individual wells of a 96-well plate. The absorbance of the reaction mixture was measured using a fluorescence microplate reader (Polarstar Galaxy, BMG LabTechnologies) at excitation and emission wavelengths of 350 and 450 nm, respectively. IC_50_ (mg/mL) was the effective concentration at which the extract exhibited 50% PL inhibitory activity as interpolated from linear regression analysis. The PL inhibitory activity was determined as follows:


$$\mathrm{Pancrelipase}\mathrm{inhibitory}\mathrm{activity}\left(\%\right)=\left[1-\frac{{\text{A}}_{1_{}}}{{\text{A}}_{0}}\right]\times 100$$

where A_1_ is the absorbance of the test sample; A_0_ is the absorbance of the blank (without samples).

#### Determination of Pancreatic Lipase Inhibition Kinetics

To identify the kinetics and pattern of extract-mediated PL inhibition, Lineweaver–Burk plots {the reciprocal of substrate concentration [1/(S)] against the reciprocal of reaction velocity [1/(V)]} were generated and the kinetic parameters (K_*m*_, K_*i*_, and V_max_) were determined using the Michaelis-Menten equation. Different concentrations of samples (0, 4.0, and 8.0 mg/mL) and substrates (0.33–4.00 mM) were prepared to measure the enzyme inhibitory activities. The L-B plots were drawn using Origin version 2021.

#### Molecular Docking Simulation

The AutoDock tool was used to examine the specific interaction between PL and major polyphenols of *P. vicina* extract (also called receptor and ligands, respectively). The PL structure (PDB ID: 1ETH) was downloaded from Protein Data Bank in the pdb format. The three-dimensional structures of the polyphenols were downloaded from PubChem database and saved in pdb formats. All the ligands and water molecules were removed within lipase using PyMOL, while all hydrogen atoms were added to the crystal structure for docking simulation. The size of the Grid box to contain the whole lipase molecule was 90 × 66 × 126 points, while the Grid spacing was 0.978. Moreover, the coordinate of Grid box center was set as follows: *x* center = 71.994, *y* center = 29.011, and *z* center = 144.799. The docking simulation programs were based on the Lamarckian genetic algorithm. AutoDock Vina was used to predict the binding sites of polyphenols in PL. The binding free energy was calculated 10 times. The conformation with the lowest binding free energy was expressed as the best conformation. Results were plotted using PyMOL, Ligplus+, and Proteins Plus.

### Statistical Analysis

All data were plotted in Origin 2021 software and analyzed by SPSS 19.0 (SPSS Inc., Chicago, IL, United States). The experimental data were expressed as means ± standard deviation (SD) of three measurements, and the results were subjected to one-way analysis of variance (ANOVA). Data with *P* < 0.05 were considered statistically significant (using the Tukey method at 95% confidence).

## Results and Discussion

### Physicochemical Parameters of *P. vicina* Extract

The moisture (12.67 ± 0.22%), protein (69.71 ± 0.51%), lipid (0.67 ± 0.09%), sugar (69.71 ± 0.51%), and ash contents (16.48 ± 0.19%), as well as TPC [28.03 ± 2.24 mg/g gallic acid equivalent (GAE)] and TFC [61.25 ± 2.55 mg/g rutin equivalent (RE)], of the *P. vicina* extract are shown in Supplementary Table 1. The TPC in the *P. vicina* extract was consistent with that reported in the *Holotrichia parallela* Motschulsky ethanolic extract (33.13 ± 1.49 mg/g GAE) (8). Moreover, the TPC in the *P. vicina* extract was higher than that in the *Allomyrina dichotoma* larval (0.33 to 18.37 mg/g GAE) and *Henicus whellani* petroleum ether extracts (0.8 g GAE/100 g), which were prepared using a Soxhlet (16, 17). The TFC in the *P. vicina* extract was higher than that reported in the extracts of various edible insects, such as *Tenebrio molitor*, *Acheta domesticus*, and *Ruspolia differens* Serville (18, 19).

### Analysis of the *P. vicina* Extract

**FIGURE 1:**
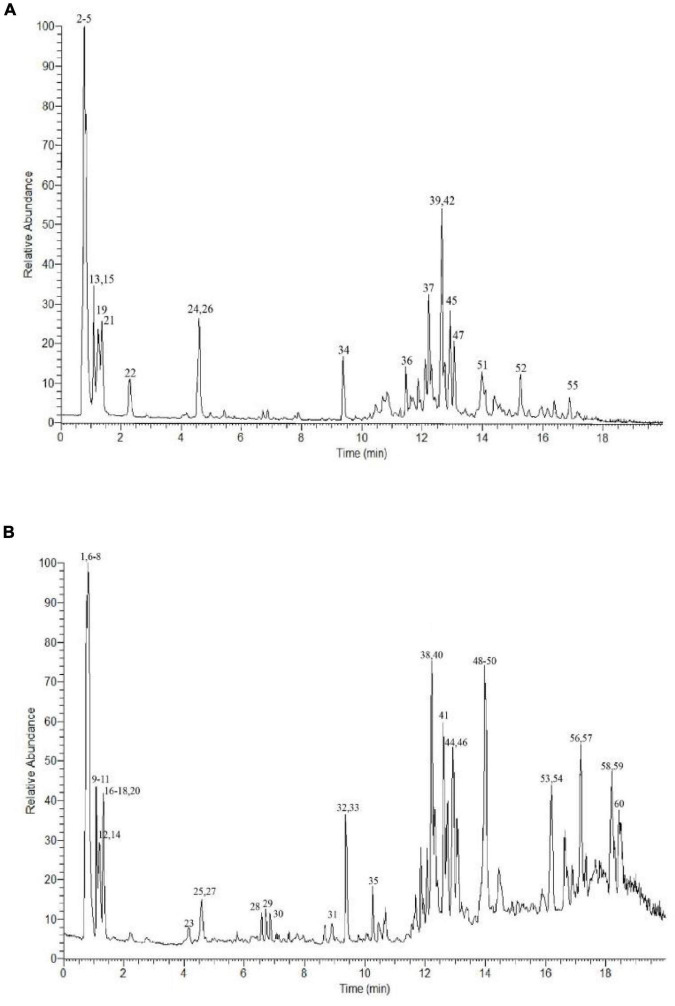
UPLC-Q-Extractive Orbitrap MS total ion chromatogram of ion mode in *P. vicina* extract. **(A)** Positive. **(B)** Negative.

The bioactive content of the *P. vicina* extract was determined according to the Human Metabolome, Metlin, MassBank, and mzCloud databases. The total ion current (TIC) diagram of the main chemical groups is shown in Figures 1A,B. In total, 60 bioactive components were detected in the *P. vicina* extract. These bioactive components were divided into the following five groups depending on their specific groups and properties: fatty acids (short-chain fatty acids and long-chain fatty acids); phenolic acids and flavonoids; organic acids; nitrogen compounds (amino acids, vitamins, and alkaloids); others. Moreover, the name, mass to charge ratio (*m/z*), retention time, type, error, *p*-value, and fragment ions of components are shown in Table 1. The internal standard used was 2-amino-3-(2-chloro-phenyl)-propionic acid.

**TABLE 1:** Characterization of bioactive components in *P. vicina* extract by UPLC-Q-Extractive Orbitrap MS.

No. peak	Rt (time)	Name	Mz	Exact_mass	Ppm	Formula	Type	Identify *p* value	Fragment ions (m/z)
1	0.69	Leucine	130.0862	131.0946	0.10	C_6_H_13_NO_2_	[M-H]^–^	0.0000	
2	0.72	L-Serine	106.0501	105.0426	1.00	C_3_H_7_NO_3_	[M+H]^+^	0.0000	60.05
3	0.73	Betaine	118.0524	117.0790	3.95	C_5_H_11_NO_2_	[M+H]^+^	0.0000	118.09, 59.03, 58.07
4	0.77	L-Isoleucine	132.1017	131.0946	0.92	C_6_H_13_NO_2_	[M+H]^+^	0.0000	86.10
5	0.80	L-Glutamic acid	148.0597	147.0532	1.96	C_5_H_9_NO_4_	[M+H]^+^	0.0000	130.05, 102.06, 84.04
6	0.80	Salicylic acid	137.0223	138.0317	0.63	C_7_H_6_O_3_	[M-H]^–^	0.0000	137.02, 121.03, 95.04, 93.03
7	0.88	Alanopine	160.0598	161.0688	1.90	C_6_H_11_NO_4_	[M-H]^–^	0.0005	88.04
8	0.89	Citramalic acid	147.0294	148.0372	3.47	C_5_H_8_O_5_	[M-H]^–^	0.0012	147.03, 129.06, 87.05, 85.06
9	1.02	Citric acid	191.0192	192.0270	2.25	C_6_H_8_O_7_	[M-H]^–^	0.0004	173.03, 111.01
10	1.07	L-Tyrosine	180.0015	181.0739	2.90	C_9_H_11_NO_3_	[M-H]^–^	0.0000	163.04, 119.05
11	1.10	Succinic acid	117.0150	118.0266	2.54	C_4_H_6_O_4_	[M-H]^–^	0.0000	99.01, 73.03
12	1.16	Isocitric acid	191.0192	192.0270	2.44	C_6_H_8_O_7_	[M-H]^–^	0.0000	191.04, 173.05, 129.04
13	1.18	L-Glutamine	147.0766	146.0691	0.23	C_5_H_10_N_2_O_3_	[M+H]^+^	0.0000	147.05, 130.05, 119.06, 84.05
14	1.20	*Trans*-Cinnamic acid	147.0452	148.0475	0.95	C_9_H_8_O_2_	[M-H]^–^	0.0000	148.05, 103.06, 77.04
15	1.22	Niacinamide	123.0552	122.0480	0.29	C_6_H_6_N_2_O	[M+H]^+^	0.0000	123.06, 96.04
16	1.25	Myo-inositol	179.0541	180.0634	3.42	C_6_H_12_O_6_	[M-H]^–^	0.0000	179.06, 161.05, 104.03
17	1.26	Adenine	134.0469	135.0545	2.27	C_5_H_5_N_5_	[M-H]^–^	0.0000	134.05, 107.04
18	1.28	Glutaric acid	131.0335	132.0423	2.14	C_5_H_8_O_4_	[M-H]^–^	0.0000	84.04
19	1.30	Guanine	152.0571	151.0494	2.83	C_5_H_5_N_5_O	[M+H]^+^	0.0000	152.06, 135.03
20	1.32	Suberic acid	173.0800	174.0892	3.12	C_8_H_14_O_4_	[M-H]^–^	0.0000	173.08, 111.08
21	1.37	L-Carnitine	162.0575	161.1052	0.15	C_7_H_15_NO_3_	[M+H]^+^	0.0000	162.11, 103.03, 85.03
22	2.34	D-Ribose	151.0353	150.0528	2.46	C_5_H_10_O_5_	[M+H]^+^	0.0006	146.03, 128.02, 100.02
23	4.12	Uridine	243.0622	244.0695	0.02	C_9_H_12_N_2_O_6_	[M-H]^–^	0.0000	200.06, 110.02
24	4.59	Pyridoxamine	169.0973	168.0899	0.77	C_8_H_12_N_2_O_2_	[M+H]^+^	0.0021	169.08, 151.07, 124.09
25	4.60	Vanillic acid	167.0350	168.0423	0.10	C_8_H_8_O_4_	[M-H]^–^	0.0000	167.03, 152.01
26	4.63	Pelargonic acid	159.0653	158.1307	0.04	C_9_H_18_O_2_	[M+H]^+^	0.0000	131.97, 113.96, 85.03, 72.94
27	4.67	D-Fructose	179.0550	180.0634	0.48	C_6_H_12_O_6_	[M-H]^–^	0.0538	179.04, 161.05, 143.03, 113.03
28	6.59	Isoferulic acid	193.0513	194.0579	1.66	C_10_H_10_O_4_	[M-H]^–^	0.0000	193.05, 178.03
29	6.66	Gallic acid	169.0141	170.0215	0.39	C_7_H_6_O_5_	[M-H]^–^	0.0000	169.01, 125.02
30	6.80	Gluconic acid	195.1222	196.0583	6.65	C_6_H_12_O_7_	[M-H]^–^	0.0000	195.05, 129.02, 99.01, 75.01
31	8.80	3,4-Dihydroxybenzoic acid	153.0193	154.0295	1.18	C_7_H_6_O_4_	[M-H]^–^	0.0006	153.02, 109.03
32	9.34	10-Hydroxydecanoic acid	187.1330	188.1412	0.67	C_10_H_20_O_3_	[M-H]^–^	0.0238	187.12, 125.10
33	9.41	Ascorbic acid	175.0239	176.0321	0.42	C_6_H_8_O_6_	[M-H]^–^	0.0647	175.02, 157.01
34	9.45	D-Tryptophan	205.0972	204.0899	0.19	C_11_H_12_N_2_O_2_	[M+H]^+^	0.0000	188.07, 146.06
35	10.27	Scopoletin	191.0341	192.0423	4.43	C_10_H_8_O_4_	[M-H]^–^	0.0075	191.03, 176.01
36	11.44	Myristic acid	229.1798	228.2089	2.27	C_14_H_28_O_2_	[M+H]^+^	0.0002	
37	11.69	Hordenine	166.1226	165.1154	0.22	C_10_H_15_NO	[M+H]^+^	0.3088	166.01, 120.08
38	12.31	Palmitoleic acid	253.2174	254.2246	0.21	C_16_H_30_O_2_	[M-H]^–^	0.0000	
39	12.35	Pantothenic acid	220.1179	219.1107	0.37	C_9_H_17_NO_5_	[M+H]^+^	0.0000	202.11, 90.06
40	12.37	Formononetin	267.0651	268.0736	2.38	C_16_H_12_O_4_	[M-H]^–^	0.0000	267.07, 253.08, 197.07
41	12.64	Palmitic acid	255.0736	256.2402	4.83	C_16_H_32_O_2_	[M-H]^–^	0.0000	255.16, 237.22
42	12.71	Undecanoic acid	186.9565	186.1620	0.02	C_11_H_22_O_2_	[M+H]^+^	0.0000	163.02, 109.03
43	12.79	Liquiritigenin	255.0800	256.0736	3.37	C_15_H_12_O_4_	[M-H]^–^	0.0177	255.10, 135.02, 119.03, 91.00
44	12.81	Quercetin	301.0352	302.0427	0.18	C_15_H_10_O_7_	[M-H]^–^	0.0001	301.04, 257.11, 179.00, 151.00
45	12.88	Riboflavin	377.1455	376.1383	0.18	C_17_H_20_N_4_O_6_	[M+H]^+^	0.0000	377.14, 243.09
46	12.88	Caffeic acid	179.0350	180.0425	0.09	C_9_H_8_O_4_	[M-H]^–^	0.0000	179.04, 135.05, 134.07
47	13.09	Naringenin	273.0763	272.0685	0.41	C_15_H_12_O_5_	[M+H]^+^	0.0548	273.10, 253.05, 153.02
48	13.80	Catechin	289.0718	290.0831	2.95	C_15_H_14_O_6_	[M-H]^–^	0.1413	289.07, 245.08, 203.07, 123.05, 109.03
49	13.85	Coumesterol	267.0295	268.0372	1.53	C_15_H_8_O_5_	[M-H]^–^	0.0000	267.05
50	13.89	Oleic acid	281.2487	282.2560	0.02	C_18_H_34_O_2_	[M-H]^–^	0.0000	
51	13.97	Gamma-Linolenic acid	279.2321	278.2246	0.78	C_18_H_30_O_2_	[M+H]^+^	0.1009	279.21, 233.18, 205.13, 149.08
52	14.70	Stearidonic acid	277.2161	276.2089	0.25	C_18_H_28_O_2_	[M+H]^+^	0.0000	277.22, 251.21
53	16.27	Sakuranetin	285.0764	286.0841	1.45	C_16_H_14_O_5_	[M-H]^–^	0.0032	285.06, 267.07, 165.02
54	16.33	Epicatechin	289.0690	290.0790	4.44	C_15_H_14_O_6_	[M-H]^–^	0.0000	289.04, 245.04
55	16.83	Nonadecanoic acid	299.2581	298.2872	0.32	C_19_H_38_O_2_	[M+H]^+^	0.0000	299.18, 184.07
56	17.25	Abscisic acid	263.1287	264.1362	0.86	C_15_H_20_O_4_	[M-H]^–^	0.3167	263.13, 219.12
57	17.30	Arachidonic acid	303.2323	304.2402	2.17	C_20_H_32_O_2_	[M-H]^–^	0.0000	303.23, 259.01
58	18.23	Octadecenoic acid	281.2487	282.2559	0.30	C_18_H_34_O_2_	[M-H]^–^	0.0000	281.25
59	18.27	Arachidic acid	311.1689	312.3028	1.67	C_20_H_40_O_2_	[M-H]^–^	0.0000	311.23
60	18.41	Docosapentaenoic acid	329.2483	330.2559	0.85	C_22_H_34_O_2_	[M-H]^–^	0.0000	329.26

#### Fatty Acids

The following 16 long-chain fatty acids were tentatively identified in the extract: glutaric acid (compound 18), suberic acid (compound 20), pelargonic acid (compound 26), 10-hydroxydecanoic acid (compound 32), myristic acid (compound 36), palmitoleic acid (compound 38), palmitic acid (compound 41), undecanoic acid (compound 42), oleic acid (compound 50), gamma-linolenic acid (compound 51), stearidonic acid (compound 52), nonadecanoic acid (compound 55), arachidonic acid (compound 57), octadecenoic acid (compound 58), arachidic acid (compound 59), and docosapentaenoic acid (compound 60). Long-chain fatty acids are the common members of the fatty acid family. Consistent with previous finding, most predominant fragment ions of long-chain fatty acids were characterized by the neutral loss of the water molecule (*m/z* 18.0) and carbon dioxide (*m/z* 44.0), which is a typical characteristic of oxocarboxylic acids (20). For example, compounds 41 and 57 exhibited [M-H]^–^ ions at *m/z* 255.16 and 303.23, respectively. The ions at *m/z* 255.16 and 303.23 resulted from the neutral loss of H_2_O (*m/z* 237.22) and CO_2_ (*m/z* 259.01), respectively. Thus, compounds 41 and 57 were characterized as palmitic acid and arachidonic acid, respectively. Additionally, oleic acid (compound 50) and arachidonic acid (compound 57), which were the main constituents of the *P. vicina* extract, are reported to exhibit various biological functions. For example, a previous study reported that oleic acid mediates the anti-obesity effect of the *P. brevitarsis* extract (21). Arachidonic acid (also named cis-5,8,11,14-eicosatetraenoic acid), a long-chain polyunsaturated fatty acid belonging to the omega-6 family, is an important constituent of cell membranes and can exert preventive and therapeutic effects on pre-eclampsia, hypertension, and diabetes mellitus (22). Zhao et al. (20) reported that the special linear structure of fatty acid molecules, including palmitoleic acid and oleic acid, prevents their dissociation into fragment ions. Additionally, the highest biochemical component in insect species or their extracts is lipids, especially free fatty acids (18).

#### Phenolic Acids and Flavonoids

The following 14 phenolic acids and flavonoids were identified in the *P. vicina* extract: salicylic acid (compound 6), *trans*-cinnamic acid (compound 14), vanillic acid (compound 25), isoferulic acid (compound 28), gallic acid (compound 29), 3,4-dihydroxybenzoic acid (compound 31), formononetin (compound 40), liquiritigenin (compound 43), quercetin (compound 44), caffeic acid (compound 46), naringenin (compound 47), catechin (compound 48), sakuranetin (compound 53), and L-epicatechin (compound 54). As phenolic acids contain a high number of carboxyl and hydroxyl groups, the fragment ions produced from these phenolic acids in the MS/MS spectra were attributed to the loss of water molecules (*m/z* 18.0) or carbon dioxide (*m/z* 44.0) in the negative ion mode. The predominant precursor ion [M-H]^–^ at *m/z* 137.02 was salicylic acid. The fragment ions of salicylic acid at *m/z* 121.03, *m/z* 95.04, and *m/z* 93.03 resulted from the loss of O, CO_2_, and CH_4_O, respectively. Meanwhile, the product ion spectrum of salicylic acid comprised *m/z* 121.03 [M-O-H]^–^, *m/z* 95.04 [M-CO_2_-H]^–^, and *m/z* 93.03 [M-CH_4_O-H]^–^. The precursor ion of gallic acid was detected at *m/z* 169.01, while the characteristic fragment ion at *m/z* 125.02 corresponded to [M-CO_2_-H]^–^. Similar fragmentation ions were also reported previously (20). Among the flavonoids, the following two characteristic fragmentation pathways can be involved: the retro-Diels-Alder reaction on the C ring and the gradual degradation of the molecule. Formononetin exhibited a protonated molecular ion [M-H]^–^ at *m/z* 267.07 and yielded major fragments at *m/z* 253.08 [M-CH_2_-H]^–^ and *m/z* 197.07 [M-CH_2_-2CO-H]^–^. Liquiritigenin generated a [M-H]^–^ ion at *m/z* 255.10, which fragmented into three product ions at *m/z* 135.02, *m/z* 119.03, and *m/z* 91.00. The characteristic fragment ions at *m/z* 135.02, *m/z* 119.03, and *m/z* 91.00 corresponded to [M-C_8_H_8_O-H]^–^, [M-C_7_H_4_O_3_-H]^–^, and [M-C_9_H_8_O_3_-H]^–^, respectively. Naringenin exhibited precursor molecular ions at *m/z* 273.10 [M+H]^+^. In the positive ion mode, the MS/MS spectra of naringenin revealed the predominant ion at *m/z* 153.02 [M-C_8_H_8_O+H]^–^. Moreover, the [M-H-28]^–^ ion observed in the fragments was attributed to the neutral loss of carbon monoxide, such as quercetin. For each analyte of phenolic acids or flavonoids, the most stable characteristic product ions rather than the most abundant product ion were selected as the product ion for quantification. Additionally, some most abundant characteristic product ions were selected as the product ions for identification to avoid false-positive results.

#### Organic Acids

UPLC-Q-Extractive Orbitrap MS analysis revealed the following seven organic acids, which are a major class of bioactive components in the *P. vicina* extract: citramalic acid (compound 8), citric acid (compound 9), succinic acid (compound 11), isocitric acid (compound 12), gluconic acid (compound 30), ascorbic acid (compound 33), and abscisic acid (compound 56). According to the dominant product ions of organic acids, the loss of ions at *m/z* 18.0 was attributed to water molecules. Compound 56 with a precursor ion at *m/z* 263.13 exhibited a major fragment at *m/z* 219.12, which was attributed to the loss of carbon dioxide (*m/z* 44.0). Isocitric acid exhibited similar fragmentation ions at *m/z* 173.05 and *m/z* 129.04. Citric acid was the major organic acid detected the organic acid profile varied depending on insect species. Previous study has reported that the predominant ion of citric acid at *m/z* 173.03 produced a fragment ion [M-CO_2_-2H_2_O-H]^–^ at *m/z* 111.01 (23). Citric acid is reported to exhibit anti-stress and immunomodulatory activities (24). For example, broilers fed with citric acid exhibited increased densities of immune-competent cells and activated immune-competent cells, namely T lymphocytes and B lymphocytes (24). The *P. vicina* extract comprised a high content of gluconic acid (compound 20). The precursor ion of gluconic acid was observed at *m/z* 195.05 with fragmented ions [M-2CO_2_-OH]^–^ at *m/z* 99.01 and [M-2CO_2_-O-OH]^–^ at *m/z* 75.01 (23). Gluconic acid, a mild non-corrosive acid, naturally occurs in fruits, plants, honey, vinegar, wine, and other natural sources (25). Limited studies have examined gluconic acid in insects. del Hierro et al. (18), for the first time, obtained a gluconic acid-rich fraction from insects (*Acheta domesticus* and *Tenebrio molitor*) using ultrasound-assisted extracted and pressurized-liquid extraction. The ultrasonic microwave method used in this study is an effective method for extracting insect gluconic acid.

#### Nitrogen Compounds

In this study, 19 nitrogen components (polar components) were identified in the extract. Of these, the following eight nitrogen compounds were identified as amino acids in positive or negative ion mode: leucine (compound 1), L-serine (compound 2), L-isoleucine (compound 4), L-glutamic acid (compound 5), alanopine (compound 7), L-tyrosine (compound 10), L-glutamine (compound 13), and D-tryptophan (compound 34). Additionally, the following four nitrogen compounds were identified as alkaloids: betaine (compound 3), L-carnitine (compound 21), scopoletin (compound 35), and hordenine (compound 37). Of the eight identified amino acids, only three were essential amino acids. The content of other essential amino acids may be low and hence could not be qualitatively determined. Thus, additional quantification of free amino acids must be performed. Compounds 15 (*m/z* 123.06, [M+H]^+^), 24 (*m/z* 169.0973, [M+H]^+^), 39 (*m/z* 220.12, [M+H]^+^), and 45 (*m/z* 377.1455, [M+H]^+^) were assigned as niacinamide, pyridoxamine (VB_6_), pantothenic acid (VB_5_), and riboflavin (VB_2_), respectively, which are water-soluble vitamins. The typical fragment ions of compounds 17 (*m/z* 107.04 [M-HCN-H]^–^), 19 (*m/z* 135.03 [M-NH_3_+H]^+^), and 23 (*m/z* 110.02 [M-C_5_H_10_O_4_-H]^–^) enabled their identification as adenine, guanine, and uridine, respectively (26). The parent ions at *m/z* 118.09, *m/z* 162.11, *m/z* 191.03, and *m/z* 166.01 were assigned as betaine (compound 3), L-carnitine (compound 21), scopoletin (compound 35), and hordenine (compound 37), respectively, based on previous reports (27–29). Compound 3 exhibited [M+H]^+^ ion at *m/z* 118.09 with the predominant product ions of *m/z* 59.03 [M-CH_2_COOH+H]^+^ and *m/z* 58.07 [M-C_3_H_9_N+H]^+^, which tentatively indicated that it was betaine (30). Compound 35 with a precursor ion [M-H]^–^ at *m/z* 191.03 and a major fragment at *m/z* 176.01 was assigned as scopoletin (27). L-carnitine exhibited a [M+H]^+^ ion at *m/z* 162.11, which fragmented into two typical product ions at *m/z* 103.03 [M-C_3_H_8_N+H]^+^ and *m/z* 85.03 [M-C_3_H_10_NO+H]^+^. Previous studies have reported that L-carnitine exerts therapeutic effects on high-fat diet-induced lipid metabolism dysfunction in C57/B6 mice (29, 31). To the best of our knowledge, this is the first study to report the production of betaine in *P. vicina*. The enrichment of amino acids and betaine was observed in the ethanol extracts of insects, except for some species, using an ultrasonic microwave method of food or healthcare products.

**FIGURE 2:**
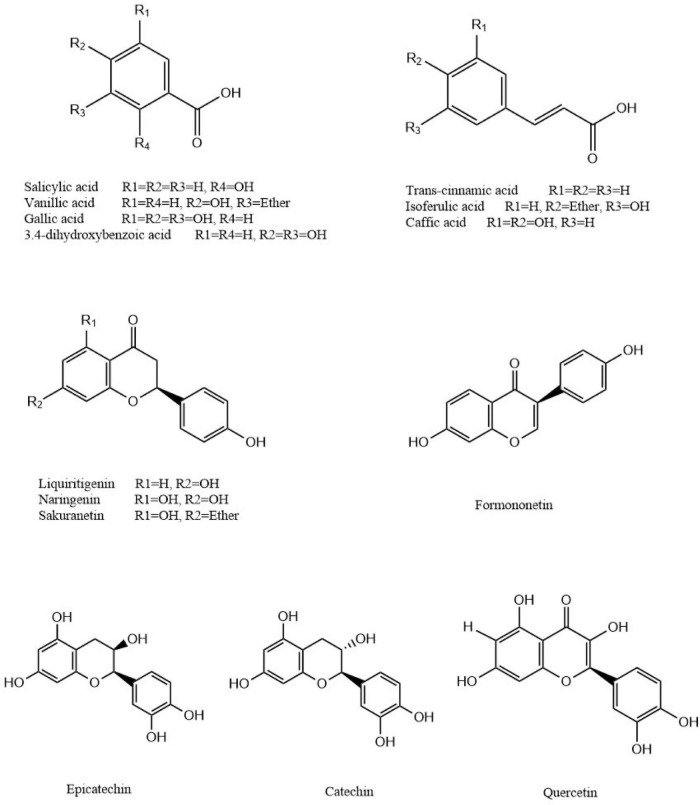
Chemical structures of polyphenols identified in *P. vicina* extract.

In total, 14 polyphenols were quantified in the *P. vicina* extract using LC-MS. The chemical structures and contents of polyphenols are shown in Figure 2 and Table 2. Additionally, the TIC and extracted ion chromatograms (EIC) in the negative mode of these compounds are shown in Supplementary Figures 1, 2. The results indicated that salicylic acid, gallic acid, liquiritigenin and naringenin were the four major polyphenols in the *P. vicina* extract.

**TABLE 2:** LC-MS characterization of fourteen polyphenols in *P. vicina* extract.

Name	Mz	Type	Contents (mg/kg)
Salicylic acid	137.0223	[M-H]^–^	593.61
*Trans*-cinnamic acid	147.0452	[M-H]^–^	50.29
Vanillic acid	167.0350	[M-H]^–^	32.28
Isoferulic acid	193.0513	[M-H]^–^	71.24
Gallic acid	169.0141	[M-H]^–^	306.04
3,4-dihydroxybenzoic acid	153.0193	[M-H]^–^	90.37
Formononetin	267.0651	[M-H]^–^	16.55
Liquiritigenin	257.0800	[M-H]^–^	224.21
Quercetin	301.0352	[M-H]^–^	60.06
Caffeic acid	179.0350	[M-H]^–^	94.49
Naringenin	273.0763	[M-H]^–^	189.96
Catechin	289.0718	[M-H]^–^	21.52
Sakuranetin	285.0764	[M-H]^–^	37.08
L-epicatechin	289.0690	[M-H]^–^	0.12

Moreover, various bioactive substances were identified through UPLC-Q-Extractive Orbitrap MS although the insect extracts were directly analyzed. The bioactive ingredients were identified using column chromatography and other advanced analytical methods owing to the limitation of the mass spectrum library and a wide variety of components. Further studies are needed to examine the composition of these extracts.

### Fatty Acid Composition of *P. vicina* Extract

As shown in Supplementary Table 2, 23 fatty acids [seven saturated fatty acids (SAFa), 12 monounsaturated fatty acids (MUFa), and 4 polyunsaturated fatty acids (PUFa)] were identified in the *P. vicina* extract. C16:0 (palmitic acid) was the predominant saturated fatty acid with a content of 3486.95 ± 8.54 μg/g of PUFa, followed by C18:0 (stearic acid) (2128.83 ± 4.88 μg/g PUFa). C18:1N9C (oleic acid) was the predominant MUFa (6765.82 ± 13.42 μg/g *P. vicina* extract), followed by C18:1N7 (vaccenic acid, 4345.05 ± 8.50 μg/g *P. vicina* extract) and C16:1 (palmitoleic acid, 1050.96 ± 6.01 μg/g *P. vicina* extract). The following four PUFas were detected in the *P. vicina* extract: C18:2n6 (linoleic acid, 953.86 ± 5.63 μg/g), C18:3n3 (alpha-linolenic acid, 297.80 ± 2.56 μg/g), C20:4n6 (arachidonic acid, 129.89 ± 3.20 μg/g), and C20:5n3 (docosapentaenoic acid, 116.78 ± 1.09 μg/g). The fatty acid composition in *P. vicina* was consistent with the results of previous studies on black ant, which reported that palmitic acid, oleic acid, and linoleic acid were the predominant fatty acids (32). The ratio of omega-6/omega-3 fatty acids in *P. vicina* extract was less than 5 (2.61), which indicated that *P. vicina* extract can be used in the food and pharmaceutical industries. Additionally, the ratio of omega-6/omega-3 fatty acids in the *P. vicina* powder was higher than five, which was not shown (0.4753/0.0907 = 5.24 g/100 g). The ratio of omega-6/omega-3 was less than 5 in other edible insects, such as *Clanis bilineata*. This ratio meets the health requirements of human intake (13). An appropriate proportion of omega-6/omega-3 fatty acids exerts various bioactivities, including anticancer, anti-atherosclerotic, antithrombotic, anti-inflammatory, and antidiabetes activities, in humans (33). Some exogenous induction methods could change the content of omega-3 fatty acids in insects by enriching the food chain. In particular, artificially adding linseed oil (rich in 57% α-linolenic acid, omega-3 fatty acids) to the feed of house crickets, lesser mealworms, and black soldier flies can increase the contents of omega-3 fatty acids in these insects (34). Additionally, the supplementation of microalgae, which are aquatic plants rich in omega-3 fatty acids (docosahexaenoic acid and α-linolenic acid), to chicken feed increased the contents of omega-3 fatty acids in eggs (35). Hence, the supplementation of food rich in omega-3 fatty acids to the feed of *P. vicina* can have beneficial effects.

### Antioxidant Activity of the *P. vicina* Extracts

The antioxidant activities of the extract were examined *in vitro* using the DPPH, ABTS, OH, and FRAP assays. A single method cannot completely reflect the antioxidant capacity of the extract owing to the complex extract composition and the differential performance of various tests. The antioxidant activities of *P. vicina* extract and powder at different concentrations are shown in Supplementary Figures 3A–D. The antioxidant activities of Vc were higher than those of others. The samples dose-dependently scavenged the free radicals. The DPPH⋅ scavenging activity of *P. vicina* extract was higher than that of *P. vicina* powder (*P* < 0.05). Additionally, the DPPH⋅ scavenging activities of *P. vicina* extract and powder were in the ranges of 30.22 ± 2.10% to 91.76 ± 0.21% and 11.04 ± 1.55% to 76.66 ± 1.86%, respectively. *P. vicina* extract exhibited the lowest IC_50_ values (0.165 mg/mL), followed by *P. vicina* powder (1.077 mg/mL). Furthermore, the ABTS⋅ scavenging activities of the *P. vicina* ranged from 35.21 ± 1.53% to 91.83 ± 0.39%. At concentrations less than 0.8 mg/mL, *P. vicina* extract exhibited higher ABTS⋅ scavenging activity than Vc. Consistent with the results of the DPPH⋅ scavenging assay, the IC_50_ value of *P. vicina* extract for ABTS⋅ scavenging was 0.123 mg/mL, which was lower than that of *P. vicina* powder (0.515 mg/mL). Moreover, the OH⋅ scavenging activity of the *P. vicina* extract (21.22 ± 2.11% to 84.80 ± 1.22%) was significantly higher than that of *P. vicina* powder (12.04 ± 0.62% to 56.66 ± 1.54%) (*P* < 0.01). Vc scavenged up to 92.74 ± 1.64% of ABTS⋅ at a concentration of 1.0 mg/mL, which indicated its potent antioxidant ability. In the FRAP assay, the reducing antioxidant power of *P. vicina* extract (2.24 ± 0.05) was significantly higher than that of *P. vicina* powder (1.45 ± 0.11) at a concentration of 4.0 mg/mL (*P* < 0.05). The significant differences between these two samples may be attributed to the increased number of bioactive components. The antioxidant activity of the extract and powder was dependent on their TPC and TFC. The chemical antioxidant activities of *P. vicina* extract varied. Consistent with the results of this study, del Hierro et al. (18) demonstrated that the antioxidant activities of the *A. domesticus* and *T. molitor* extracts were correlated with the TPC values. Similarly, a strong correlation between antioxidant activities and TPC or TFC has been reported in other studies. In addition to TPC and TFC, the contents of amino acids and peptides also contribute to the antioxidant activity (36, 37). However, the antioxidant activity of the extracts of different insect species has not been previously attributed to specific components (18).

### Pancreatic Lipase Inhibitory Activity and Kinetics of the Insect Extracts

**FIGURE 3:**
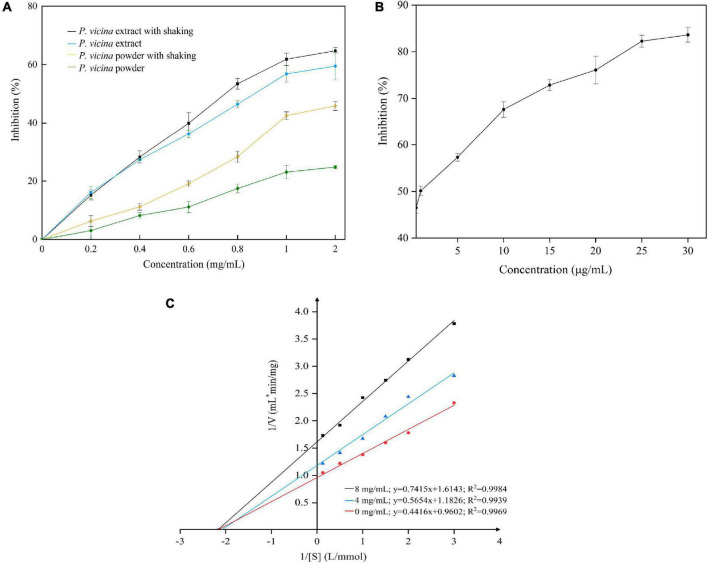
Inhibition of PL by *P. vicina* extract **(A)** and orlistat **(B**, without shaking), and the Lineweaver–Burk plot of the reaction of PL and *P. vicina* extract **(C)**.

The inhibition of PL mitigates excessive fat deposition in the adipose tissue (3). The results of PL inhibitory activity of the *P. vicina* extract with or without shaking are shown in Figure 3A. *P. vicina* extract and powder dose-dependently inhibited PL activity. The PL inhibitory activity of the *P. vicina* extract was higher than that of the *P. vicina* powder. Meanwhile, orlistat also has a significant concentration dependence on PL inhibitory activity, and the result is illustrated in Figure 3B. The estimated IC_50_ values of *P. vicina* extract with or without shaking were 0.833 mg/mL and 1.031 mg/mL, whereas that of orlistat without shaking was 1.053 μg/mL. This indicated that *P. vicina* extract had lower PL inhibitory effectiveness than orlistat. However, the specific activity of the *P. vicina* extract was higher than that of other PL inhibitors isolated from different plants. For example, the IC_50_ values of “Carolea” of *Olea europaea* extract, and *Salvia miltiorrhiza* Bunge extract for PL inhibition were 1.27 ± 0.04, and 3.54 ± 0.22 mg/mL, respectively (38, 39). Additionally, shaking did not significantly affect the PL inhibitory activities of the *P. vicina* extract (*P* > 0.05). In contrast, shaking markedly affected the PL inhibitory activities of *P. vicina* powder (*P* < 0.05). At a concentration of 2.0 mg/mL, the PL inhibitory activities of *P. vicina* extract or powder subjected to shaking were higher than those not subjected to shaking, which was consistent with the results of a previous study (15). This suggested that shaking might promote adequate dispersion of the extract in the solution and the binding of the extract to the active site of lipase, which will result in enhanced interaction with the substrate.

In this study, the *P. vicina* extract significantly inhibited the activity of PL. The specific components of insects associated with the PL inhibitory activity are complex. The PL inhibitory activities of most natural materials are attributed to various bioactive components, such as polyphenols, saponins, chitosan, or terpenes. According to the double reciprocal equation ($\frac{1}{\text{V}}=\frac{\text{Km}}{\text{Vmax}}\,\frac{1}{\text{S}}+\frac{1}{\text{Vmax}}$), the Lineweaver–Burk plot is shown in Figure 3C. The *P. vicina* extract dose-dependently decreased the V_max_ of reaction (V_max_ = 1.04, 0.84, and 0.61 at concentrations of 0.0, 4.0, and 8.0 mg/mL, respectively) but did not affect the Michaelis constant K_m_ (K_m_ = 2.15). The constant K_m_ and decreased V_max_ values indicated that the *P. vicina* extract non-competitively inhibited PL. Thus, the ability of PL to bind to the substrate 4-MUO was independent of the concentration of the extract. Fang et al. (40) reported that Monascus-fermented rice non-competitively inhibited lipase.

### Molecular Docking of the Interaction

**FIGURE 4:**
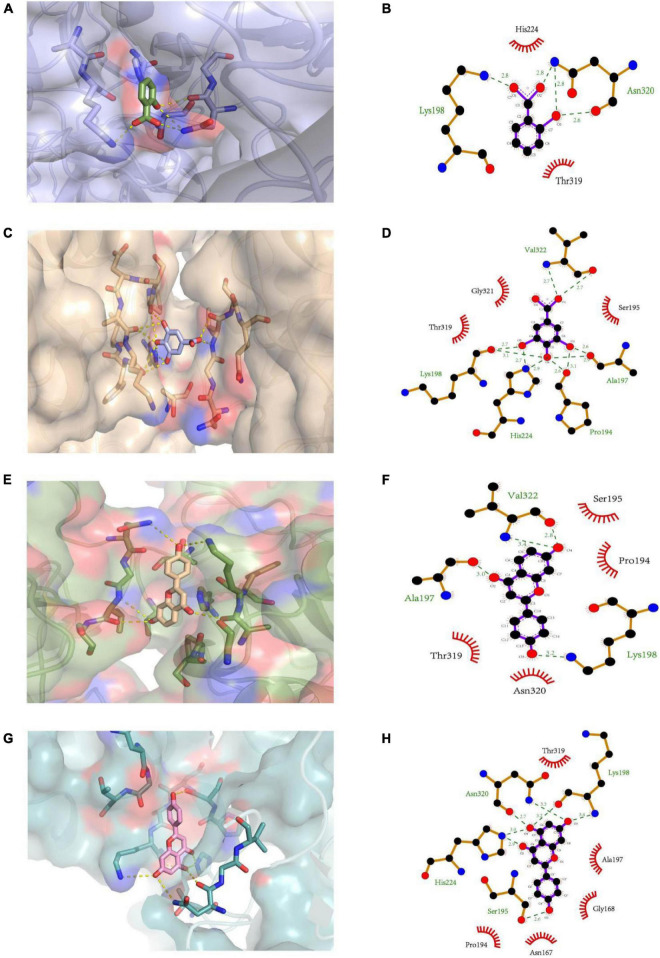
The 3D structure of **(A)** salicylic acid, **(C)** gallic acid, **(E)** liquiritigenin, and **(G)** naringenin docking with PL, respectively. The 2D schematic diagram of **(B)** salicylic acid, **(D)** gallic acid, **(F)** liquiritigenin, and **(H)** naringenin interacting with PL, respectively.

The molecular docking simulations of the interactions between PL and ligands (salicylic acid, gallic acid, liquiritigenin, and naringenin) are illustrated in Figure 4. Salicylic acid exhibited hydrogen bonding with the oxygen and nitrogen atoms of Lys198 and Asn320 (three combing sites) in PL at distances of 2.8, 2.6, 2.8, and 2.8 Å. Additionally, the imidazole group of His224 in PL may be involved in a pi-cation interaction with the cyclic structure of salicylic acid. However, salicylic acid interacted with other key amino acid residues (His224 and Thr319) of PL through hydrophobic interactions. The four oxygen atoms from the α-carboxylic acid groups of Lys198 (two combining sites), Ala197 (two combining sites), Pro194 (two combining sites), and Val322 in PL formed hydrogen bonds with the hydrogen atoms from the hydroxyl and carboxyl groups of gallic acid at distances of 2.7, 3.1, 2.6, 2.9, 2.6, 3.1, and 2.7 Å. Meanwhile, the side-chain nitrogen atoms from the imidazole group of His224 (two combining sites) formed hydrogen bonds with one nitrogen atom of Val322 in PL at distances of 2.7, 2.9, and 2.7 Å. Gallic acid interacted with three key catalytic amino acid residues (Gly321, Thr319, and Ser195) of PL through hydrophobic interactions. The two oxygen atoms from the Ala197 and Val322 α-carboxylic acid groups in PL exhibited hydrophilic interactions with the hydrogen atoms in liquiritigenin at distances of 3.0 and 2.8 Å, respectively. Additionally, the nitrogen atoms from Val322 and Lys198 (position sixth) in PL exhibited hydrophilic interactions with the hydrogen atoms of the ligand at distances of 3.4 and 3.2 Å, respectively. In addition to the hydrophilic interactions with the amino acid residues (Ser195, Pro194, Thr319, and Asn320) of PL, liquiritigenin interacted with the cyclic Pro in PL through pi-cation interaction. Naringenin exhibited the most complicated interactions. Four oxygen atoms from the α-carboxylic acid group of Ser195, Lys198, and Asn320 in PL exhibited hydrophilic interactions with the hydrogen atoms in naringenin at distances of 2.6, 3.2, and 2.7 Å, while the one nitrogen atom from the imidazole group of His224 (two combining sites), Lys198 (position sixth), and Asn320 (position forth) exhibited hydrogen bonding with PL at distances of 2.9, 3.0, 3.4, and 3.3 Å. Additionally, naringenin exhibited strong hydrophobic interactions with the hydrophobic residues, including Thr319, Ala197, Gly168, Asn167, and Pro194, of PL.

Furthermore, orlistat interacted with the amino acid residue (Ser153) at the active site of PL through a conventional hydrogen bond at a distance of 2.7 Å (41). The findings of this study suggest that similar to orlistat, polyphenols may function as PL inhibitors by competing with the substrate for the active site of PL (42). Currently, the relationship between docking results, mode of interaction, sites, and inhibitory activity is unclear. These interactions could not significantly alter the secondary structure of PL.

## Conclusion

In total, 60 bioactive components were identified in the hydro-ethanolic extract of *P. vicina* using UPLC-Q-and naringenin and 23 free fatty acids (including palmitic acid, palmitelaidic acid, stearic acid, petroselinic acid, oleic acid, vaccenic acid, and linoleic acid) were initially quantified as the major components. Furthermore, molecular docking analysis revealed that salicylic acid, gallic acid, liquiritigenin, and naringenin interacted with PL through hydrogen bonding and hydrophilic or hydrophobic and pi-cation interactions. The potent antioxidant and PL inhibitory activities of *P. vicina* extract indicated that it can be used as a nutraceutical to alleviate oxidative stress-induced disease and treat obesity.

## Data Availability Statement

The original contributions presented in the study are included in the article/Supplementary Material, further inquiries can be directed to the corresponding authors.

## Author Contributions

ZZ conceived the idea and wrote the manuscript. SC and XW supported the data analysis. JX and DH revised the manuscript. All authors contributed to the article and approved the submitted version.

## Conflict of Interest

The authors declare that the research was conducted in the absence of any commercial or financial relationships that could be construed as a potential conflict of interest.

## Publisher’s Note

All claims expressed in this article are solely those of the authors and do not necessarily represent those of their affiliated organizations, or those of the publisher, the editors and the reviewers. Any product that may be evaluated in this article, or claim that may be made by its manufacturer, is not guaranteed or endorsed by the publisher.
