# Supplementary material for: Characterization, Antioxidant Activities, and Pancreatic Lipase Inhibitory Effect of Extract From the Edible Insect Polyrhachis vicina
Source: Front Nutr. 2022 Apr 7;9:860174. doi: 10.3389/fnut.2022.860174 (PMC9021923; doi:10.3389/fnut.2022.860174)
Supplement: Supplementary file 1 [file Data_Sheet_1.doc]

Supplementary Material

**Supplementary Table 1. The chemical composition of *P. vicina* extract**

| Chemical composition | *P. vicina* extract |
| --- | --- |
| Moisture (%) | 12.67 ± 0.22 |
| Protein (%) | 69.71 ± 0.51 |
| Lipid (%) | 0.67 ± 0.09 |
| Sugar (%) | 69.71 ± 0.51 |
| Ash (%) | 16.48 ± 0.19 |
| Total polyphenolic (mg GAE^a^ /g extract) | 28.03 ± 2.24 |
| Total flavonoid (mg RE^b^ /g extract) | 61.25 ± 2.55 |

^a^Gallic acid equivalent

^b^Rutin equivalent

**Supplementary Table 2.** GC-MS characterization of fatty acid contents in *P. vicina* extract

| Name | Tr (min) | Contents (μg/g) |
| --- | --- | --- |
| C12:0 | 8.76 | 30.51 ± 1.05 |
| C14:0 | 10.58 | 127.28 ± 2.31 |
| C14:1T | 11.16 | 34.11 ± 0.95 |
| C14:1 | 11.46 | 34.54 ± 0.22 |
| C16:0 | 12.94 | 3486.95 ± 8.54 |
| C16:1 | 13.84 | 1050.96 ± 6.01 |
| C17:0 | 14.33 | 50.48 ± 0.62 |
| C17:1 | 15.29 | 91.02 ± 1.65 |
| C18:0 | 15.83 | 2128.83 ± 4.88 |
| C18:1N9T | 16.45 | 23.74 ± 0.60 |
| C18:1N7T | 16.52 | 55.36 ± 0.79 |
| C18:1N12 | 16.65 | 799.83 ± 2.56 |
| C18:1N9C | 16.71 | 6765.82 ± 13.42 |
| C18:1N7 | 16.84 | 4345.05 ± 8.50 |
| C19:1N9T | 18.05 | 295.18 ± 3.11 |
| C18:2N6 (n=6) | 18.14 | 953.86 ± 5.63 |
| C20:0 | 19.09 | 111.15 ± 1.01 |
| C20:1 | 19.92 | 243.51 ± 4.22 |
| C18:3N3 (n=3) | 20.04 | 297.80 ± 2.56 |
| C22:0 | 23.22 | 44.54± 0.60 |
| C22:1N9 | 24.45 | 39.22 ± 0.88 |
| C20:4N6 (n=6) | 24.45 | 129.89 ± 3.20 |
| C20:5N3 (n=3) | 27.51 | 116.78 ± 1.09 |
| Total Saturated |  | 5979.74 ± 15.72 |
| Total Monounsaturated |  | 13778.34 ± 18.35 |
| Total Polyunsaturated |  | 1498.33 ± 7.50 |

**
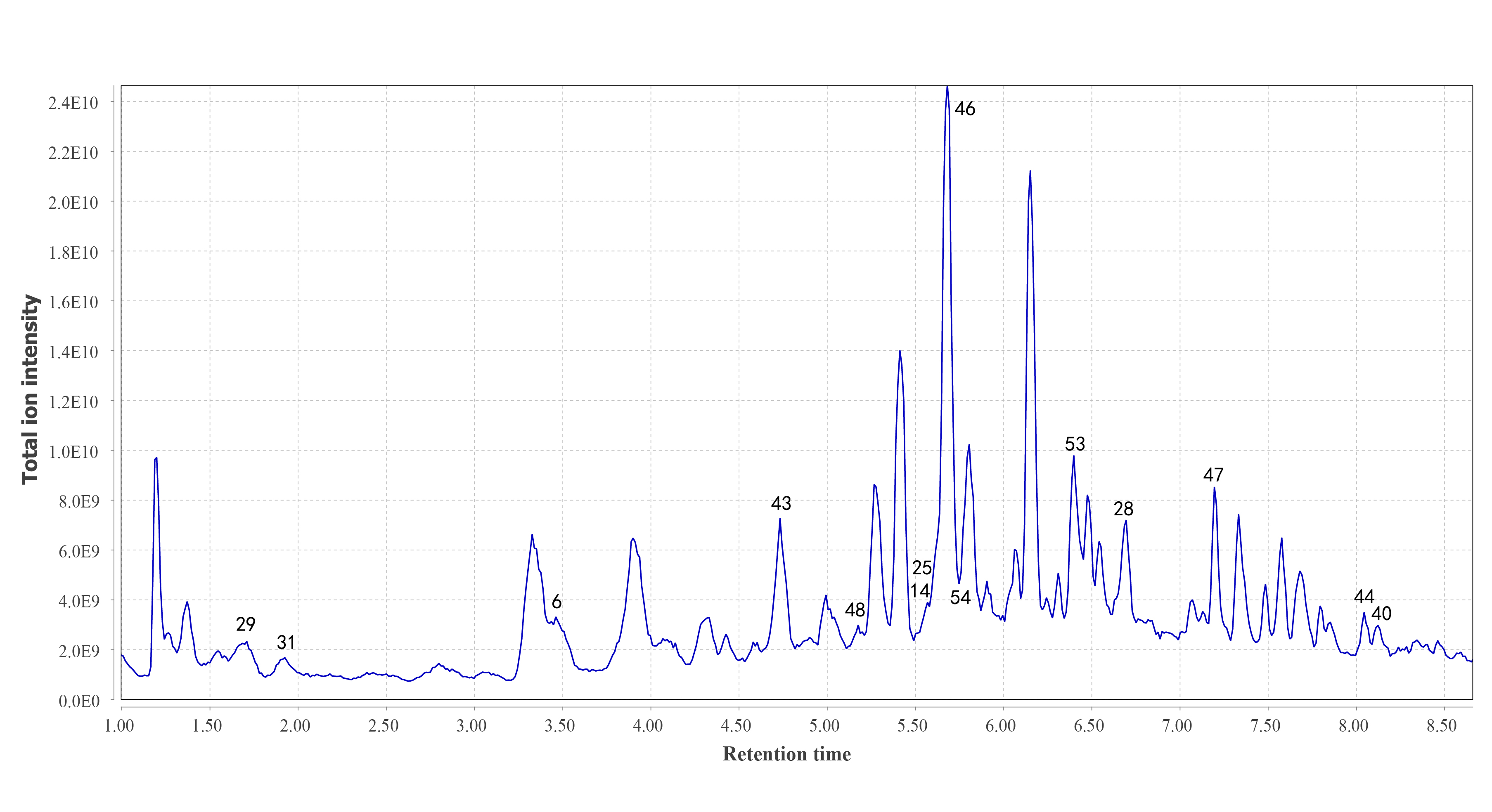
**

**Supplementary Figure 1.** Total ion chromatogram (TIC) of the 14 polyphenols in *P. vicina* extract. 14. trans-cinnamic acid, 6. salicylic acid, 28. isoferulic acid, 25. vanillic acid, 29. gallic acid, 31. 3,4-dihydroxybenzoic acid, 46. caffeic acid, 54. L-epicatechin, 43. liquiritigenin, 47. naringenin, 48. catechin, 44. quercetin, 40. formononetin, 53. sakuranetin


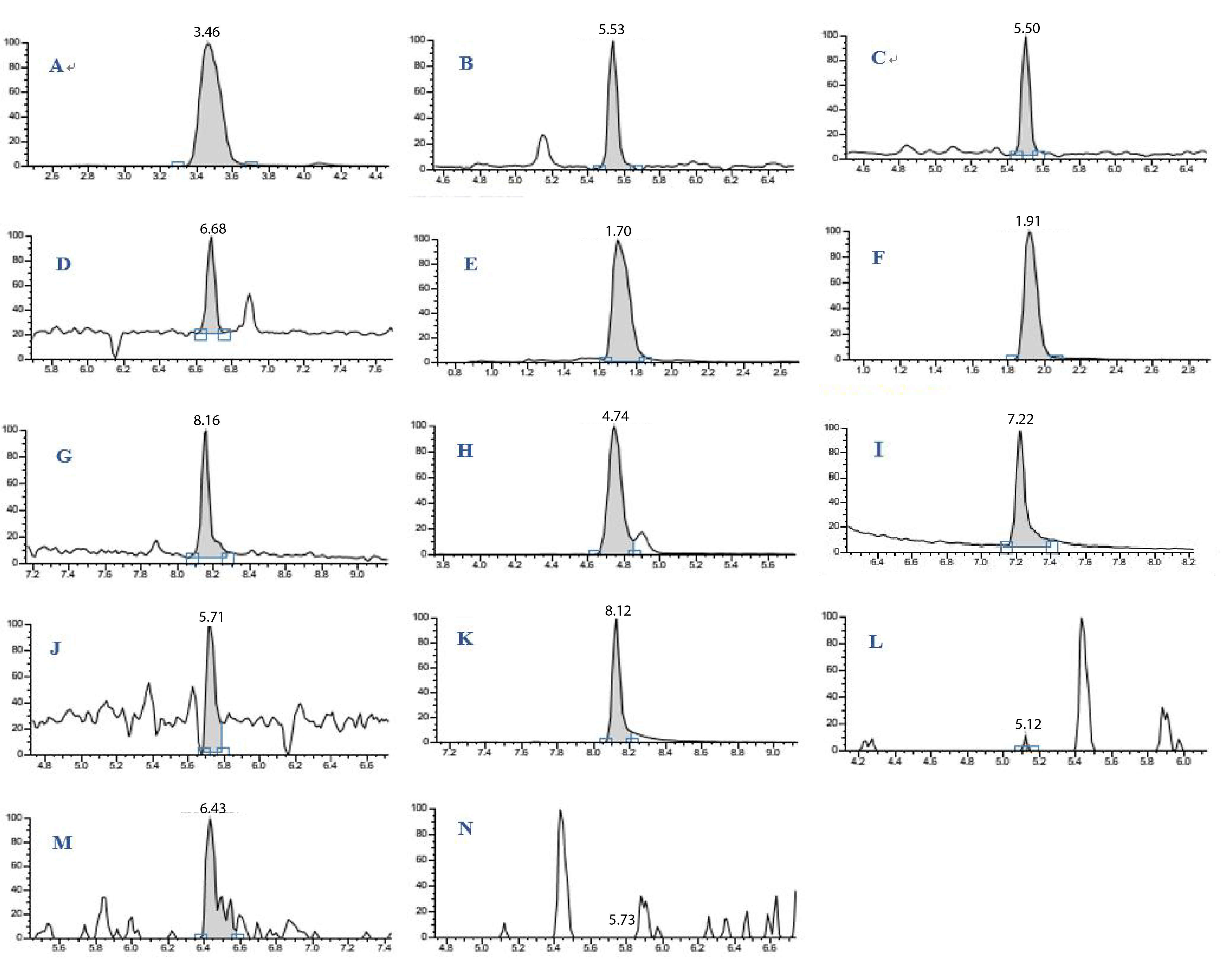


**Supplementary Figure 2.** Extracted ion chromatograms (EIC) of the 14 polyphenols in *P. vicina* extract. **(A)** salicylic acid. **(B)** trans-cinnamic acid. **(C)** vanillic acid. **(D)** isoferulic acid. **(E)** gallic acid. **(F)** 3,4-dihydroxybenzoic acid. **(G)** formononetin. **(H)** liquiritigenin. **(I)** naringenin. **(J)** caffeic acid. **(K)** quercetin. **(L)** catechin. **(M)** sakuranetin. **(N)** L-epicatechin


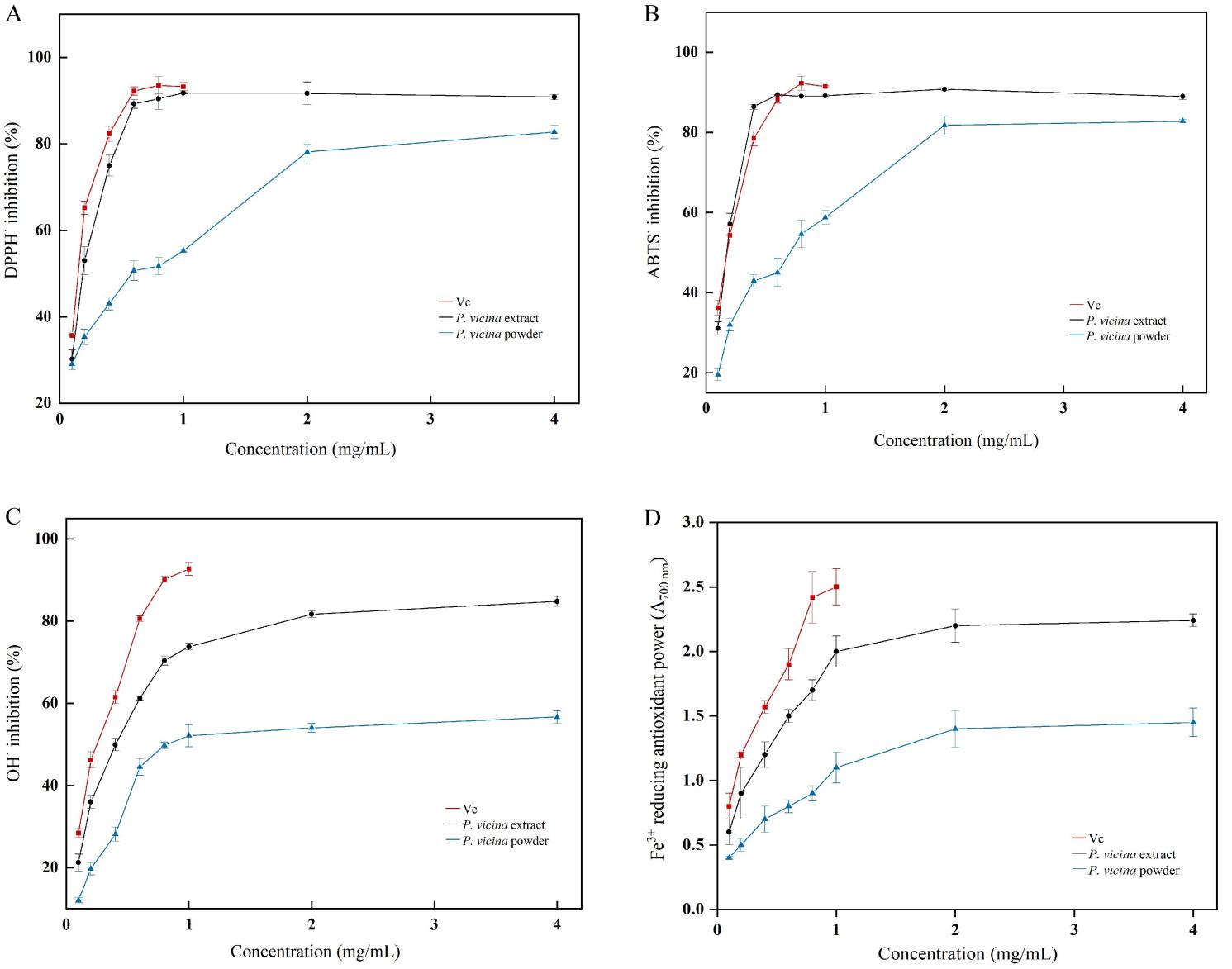


**Supplementary Figure 3.** Antioxidant assays of *P. vicina* extract. **(A)** DPPH˙. **(B)** ABTS˙. **(C)** OH˙. **(D)** Fe^3+^ reducing antioxidant power.
